# An integrated support model for lay health care workers to improve clients’ retention in HIV care

**DOI:** 10.4102/safp.v67i1.6115

**Published:** 2025-08-13

**Authors:** Sarah B. Pitse, Patrone R. Risenga

**Affiliations:** 1 Department of Health Studies, College of Human Sciences, University of South Africa, Pretoria, South Africa

**Keywords:** lay healthcare workers, healthcare workers, retention in HIV care, instrumental support, emotional support, support model, mutual dependence, communication

## Abstract

**Background:**

Retention in care is vital for the successful management of human immunodeficiency virus (HIV). About 20% of clients interrupt their HIV therapy within 6 months of starting it. Lay healthcare workers complement the healthcare professionals to provide services across the HIV care continuum. However, there is limited support for lay healthcare workers as compared to healthcare professionals. Therefore, this study was conducted at a primary healthcare centre in South Africa to develop a support model for lay healthcare workers to improve clients’ retention in HIV care.

**Methods:**

Interviews were conducted with the lay healthcare workers, their supervisors, nurses and clients on antiretroviral therapy. The support model was then developed by following Dickoff’s survey list of a theoretical framework and the theory development processes of Chinn et al.

**Results:**

The context, agent, recipient, dynamics, procedure and outcomes of support were described in the support model. To nurture lay healthcare workers, the nurses, supervisors and department of health must provide a good environment, recognise each person’s needs, offer both practical and emotional support, encourage mutual reliance and provide feedback.

**Conclusion:**

Support is an ongoing, mutually beneficial action that is essential to the efficient operation of lay healthcare workers.

**Contribution:**

Improved motivation, job satisfaction, competence and thriving are possible outcomes of the support for lay healthcare workers. This would increase the number of clients retained in HIV care, improve viral suppression and reduce transmission.

## Introduction

The intrinsic roles of various lay healthcare workers (LHWs) in human immunodeficiency virus (HIV) care and the ability to retain clients in care facilitate HIV programme outcomes.^1^ South Africa needs to be prioritised in pursuing urgent and intensive interventions because it accounts for 20% of the global HIV cases.^2^ In addition, the healthcare facilities still receive severely ill clients because of seeking clinical care late, discontinuing treatment or not starting antiretroviral therapy (ART).^3,4^ The 20% and 30% of clients who stopped ART within 6 and 12 months, respectively, and a further 26% who stopped treatment at least once, highlight the retention issues in South Africa.^3,5^

It is crucial to keep clients in HIV care because, as they take ART, the virus is suppressed, thereby lowering the risks of new infections, illness and death.^1,6,7^ Furthermore, ongoing care will prevent high ART expenditures associated with treating HIV variants that are resistant to first-line therapy. While community healthcare workers (CHWs) are essential to HIV care, Mundeva et al. found that their duties are inadequately incorporated into the healthcare system.^8^ Consequently, there could be a decline in the standard of services provided. Likewise, the Joint United Nations Programme on HIV/AIDS (UNAIDS) cautioned that because HIV care is delivered on a continuum, the operation of role actors in isolation and without direction would result in undesirable HIV outcomes.^9^ Also, Kok et al. stated that the LHWs’ lack of credibility could be attributed to the insufficient support provided to them.^10^

Prior research primarily examined the responsibilities and experiences of lay counsellors and CHWs, leaving out additional categories of LHWs such as tracers and health promoters. Nevertheless, some lay counsellors and CHWs felt supported, while others did not, and this highlighted the differences in individuals’ support perceptions.^10,11^ Support was emphasised by Sam-Agudu et al., who stated that the incorporation and acknowledgement of LHWs were essential to maximise their impact in healthcare^12^; and Engelbrecht et al., who advocated for ongoing training, support, teamwork and connection of home-based caregivers to healthcare facilities.^13^ To further enhance the HIV programme outcomes, research on the effects of organisational and environmental factors on the LHWs’ work, including their interaction with clients and co-workers, needs to be considered.^14^ Also, the fact that LHWs received less formal training than healthcare professionals makes their support critical.^15^ The study therefore sought to establish an integrated support model for LHWs to improve clients’ retention in HIV care.

## Research methods and design

The study was carried out in two stages, with careful consideration of the ethical principles and criteria for rigour. The first stage encompassed the study design, methodology and data gathering techniques. The study was non-experimental and employed an exploratory, descriptive and contextual design along with a qualitative approach. It was conducted at a community health centre in the Bojanala district of the North-West Province of South Africa. Based on the Three Interlinked Electronic Register.net (TIER.net), Bojanala district had the largest ART programme in the province, with over 7000 clients still on ART at the chosen health centre at the end of January 2021.^16^

The study population consisted of the LHWs, their supervisors and nurses (collectively termed and used interchangeably with ‘healthcare professionals’ in this study) and clients on ART. To accomplish the research objectives, three groups of participants were purposively sampled, that is, (1) LHWs who interact with persons living with HIV; (2) healthcare professionals who oversee, evaluate and know the roles of LHWs; (3) clients who have been receiving ART for a minimum of a year. With the exception of clients, who were identified in the consultation rooms, participants were identified at the manager’s office, and appropriate times for interviews were thereafter arranged with those who consented. The final group samples, as steered by data saturation, were as follows: 22 LHWs who had four focus group discussions, 10 healthcare professionals and 15 clients on ART who had in-depth interviews.^16^

With consent, data were gathered in the individuals’ native tongue, recorded and then transcription was performed in English. After conducting a thematic analysis of the data, the integrated support model, which is the subject of this article, was established. The second stage of the study began with the concept analysis, then proceeded to the structure to finalise the intended support model. The support model was created using the conceptual framework, findings from participants, and literature.^16^ The purpose of this paper is to disseminate the support model’s findings.

### Model development

The four stages of theory development – concept analysis and classification, relationship statement construction, model description, and critical reflection that Chinn et al. presented – served as the foundation for the support model.^17,16^ Following Walker and Avant’s eight processes, the first step of concept analysis examined ‘support’. These processes include: (1) choosing a concept; (2) defining the purpose of the analysis; (3) identifying all potential applications for the concept; (4) identifying its defining attributes; (5) building a model case; (6) building an additional case; (7) identifying the concept’s antecedents and consequences; and (8) defining the concept’s empirical referents.^18,19,20^ However, this paper does not discuss concept analysis in detail, but focuses on the concept classification, construction of relationship statements, model description and reflection as proposed by Chinn et al.^17^

### Ethical considerations

An application for full ethical approval was made to the University of South Africa’s College of Human Sciences Research Ethics Review Committee and ethics consent was received on 21 May 2021. The ethics approval number is 44434960_CREC_CHS_2021. The North-West Health Research, Monitoring and Evaluation Directorate; Bojanala health district; Rustenburg sub-district and the operational manager of the selected health centre were consulted for permission. Subsequently, consent was obtained from all three participant groups, who were also informed of their right to decline or discontinue participation at any moment without giving a reason or fear of penalties. The participants’ privacy and anonymity were promoted by keeping their information confidential and storing it in a locked cabinet and password-protected laptop.

## Results

### Classification of concepts

The 1968 survey list, as developed by Dickoff et al., served as the basis for the categorisation of concepts pertaining to support.^21^ Contained in the list are six items: the context, agent, recipient, dynamics, procedure, and terminus or outcome.

#### Context

The setting, environment or circumstance in which activities take place is known as the context. The study identified two contexts as shown in Figure 4^16^: context 1 as the guidelines or policies that direct the work of LHWs, namely, the national policy on HIV testing services^22^ and guidelines on management of HIV in adults, adolescents, children and infants and prevention of mother-to-child transmission;^23^ adherence for HIV, tuberculosis (TB), and non-communicable diseases;^24^ and policy framework and strategy for ward-based primary healthcare outreach teams^25^; context 2 as the healthcare facilities and community spaces where LHWs carry out responsibilities such as HIV testing and counselling, health education and adherence support, household registrations, as well as tracking and tracing of clients who interrupted therapy.^16^

The participants mentioned some guidelines or tools below^16^:

‘We empower LHWs in terms of giving them the updates because we all have to follow the updated adherence guidelines, consolidated HIV guidelines and other available standard procedures.’ (T10HP10, 46-years-old, female, health care professional)

‘So far, I have communicated with one counsellor supervisor to assist with the kids’ disclosure and adherence plan gaps as she visits the facilities. She promised to provide information on the adherence plan and emphasise that it must be used for all clients that are starting ART, unsuppressed or returning after interrupting treatment.’ (T10HP10, 46-years-old, female, health care professional)

‘The OTLs also accompanied them to the households to assess the skills against the standard checklist, identified the gaps and used the teachable moments, meaning that they addressed the gaps immediately.’ (T9HP9, 57-years-old, female, health care professional)

#### Agent

**FIGURE 1:**
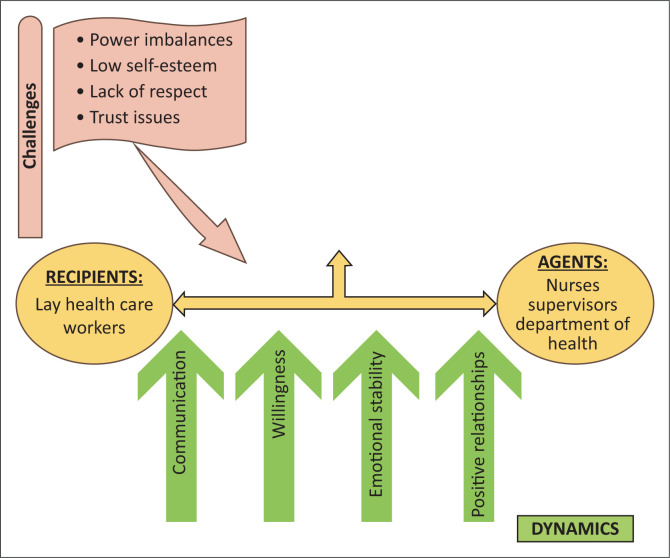
The recipients, agents and dynamics that drive support.

An agent is an individual or group of people designated to perform a certain task. So, in this study, the LHWs identified healthcare professionals and health department as the main sources of practical assistance and emotional support, as illustrated in Figure 1. To offer emotional and practical assistance to the LHWs, healthcare professionals must be competent, motivated, interested, receptive and emotionally stable.^16,26,27^

Although the first quote below is from a LHW who indicated that they support each other but lack supervisory support, the subsequent quotes are from healthcare professionals, highlighting their willingness, assistance and challenges towards LHWs’ support^16^:

‘Support, no. The managers never call us to ask about our challenges. Instead, we support each other when we meet difficult cases.’ (T3F3B, 48-years-old, male, lay health care worker)

‘In most cases when the CHWs bring patients back to the clinic, I avail myself to assist so that these patients do not wait for a long period of time or are moved up and down. Also, when CHWs are out in the field, they are able to call and discuss matters with me where necessary. I then assist them where I can, that is how I support them. When I identify that they lack knowledge, I train them or arrange with the relevant personnel to train them.’ (T3HP3, 46-years-old, female, health care professional)

‘As I visit the lay counsellors at their facilities, I discuss with them, identify gaps and address these gaps through mentoring.’ (T7HP7, 31-years-old, male, health care professional)

‘The challenge is that the 2020 CHWs have not had training, and because of COVID-19, I believe there hasn’t been enough time to properly orient them to the program, tasks, and expectations accordingly.’ (T9HP9, 57-years-old, female, health care professional)

‘In addition to accompanying them to clients for treatment, I also go to clients who have questions that CHWs are unable to answer. I then explain in a way that clients will understand and also touch on the importance of taking treatment correctly. Like questions from women of child-bearing age who want to conceive.’ (T1HP1, 58-years-old, female, health care professional)

‘I think their stipend must be increased; they are doing a good job. And occasionally, let them be transported, especially those who visit hard-to-reach areas, those working locally are okay. And then I wish that in their contract, they can have overtime allowance, for example, if a patient can only be found on Saturday, the CHW can go there on that day and be paid for overtime.’ (T9HP9, 57-years-old, female, health care professional)^16^

#### Recipient

An individual or people who stand to gain from obtaining a specific service are referred to as the recipient(s). In this study, the different categories of LHWs, namely, lay counsellors, health promoters, tracers and CHWs, who would potentially improve their retention efforts if supported adequately, are regarded as recipients. While the agent offers assistance, the support process, as depicted in Figure 1, is interactive and necessitates the recipient’s active contribution as well.^16^ In addition to being interested and emotionally secure, the recipients must recognise and express their need for help. The LHWs indicated that they needed assistance with the following: equitable work material distribution; training on pertinent skills and current HIV information; supervision and mentoring on client care; activity allocation and balancing; debriefing and discussion of obstacles; communication and cooperation; as well as improved work contracts and appreciation.

Below are some quotations from LHWs^16,20^:

‘On the other hand, supervisors are good at providing working material, yes, they are trying their best in this regard.’ (T1F1B, 34-years-old, male, lay health care worker)

‘I need a refresher training on adherence counselling because I was trained long time ago when I was a counsellor at another province, that is why I do not even know the adherence tools used. In service training must be a continuous thing and not done once. Other HIV-related topics must also be discussed so that we can gain the necessary skills and have the updated information.’ (T4F4C, 33-years-old, female, lay health care worker)

‘The other challenge is our daily HIV testing target. It used to be 8 but now it is 12 and we work for 6 hours due to our contract (other counsellors concurred). So, we end up striving to reach the target before we knock off, at the same time losing focus on other tasks.’ (T1F1C, 34-years-old, female, lay health care worker)

‘We really want to work and do the correct things, but we are sad. How can we work effectively when we are sad? It is not possible. Our supervisor also does not support us. We feel lost, the problem is that when we started to work as lay counsellors we were volunteering. Even now we are still treated as volunteers and volunteers do not have any form of support.’ (T3F3A, 37-years-old, female, lay health care worker)^16,20^

‘So, we were hoping that the ratio of 1:6 of OTL (outreach team leader) and CHWs would work because it is a manageable number so each OTL will meet with his/her CHWs and provide guidance.’ (T2F2C, 44-years-old, female, lay health care worker)

#### Dynamics

The dynamics are what propel the activity forward and are shown together with the agent and recipient in Figure 1. Building positive relationships, communicating, being willing and having stable emotions are all necessary for receiving or supporting someone. These aspects encourage cooperation, respect, trust and the unrestricted engagement of workers, paving the way for support.^16^ However, the difficulties that LHWs face discourage them from doing their best, which threatens their ability to facilitate retention in HIV care. These issues include power imbalances and low self-esteem because of the healthcare professionals’ superiority over the LHWs and no formal recognition of LHWs, all of which have a detrimental effect on communication. Other issues include a lack of respect and trust, which obstructs constructive relationships and teamwork.

Quotations below highlight the participants’ perspectives^16,20^:

‘When she mentioned communication, she has wrapped it up. Communication is the main one.’ (T2F2C, 44-years-old, female, lay health care worker)

‘I picked up that the CHWs do not even have a good relationship with their immediate supervisors. I am saying this because some of them come to me when they have a problem, but I expect them to go to their immediate supervisor. If I can give you an example, there is a new resolution that the CHWs must sign the duty register daily. Some of them still forget because it is a new thing, and I am not saying that it is right for them not to sign, all I am saying is that they need to be helped to come up with a reminder system until they get used to it. One of them came to me crying because her supervisor made them (all CHWs) sign leave without pay. I feel that this is harsh and could have been handled differently. Again, it may negatively affect the working relationship.’ (T4HP4, 50-years-old, female, health care professional)

‘It will affect their work negatively because if the CHWs are struggling with something related to work, they may not ask for assistance from their supervisor because they are afraid of her. It means they’ll continue either doing the wrong thing or not doing that task at all. They are not comfortable, so it means they’ll just work without a positive impact.’ (T4HP4, 50-years-old, female, health care professional)

‘It angers me because they expect miracles from us. And no matter how hard we work, no one appreciates or commends us for our hard work (Other participants agreed: yes). Instead, they keep on putting pressure on us, they complain and complain. And when they do this, we end up being demotivated and discouraged to work, what is the use of trying to do your best when no one notices or appreciates? A “thank you” once in a while will do.’ (T2F2A, 33-years-old, female, lay health care worker)^16,20^

#### Procedure

Five steps are used to summarise the ongoing process of support^16^:

**Step 1:** Create a positive climate: Supportive actions begin with cultivating a good environment where LHWs can feel free to express their opinions. To foster positive connections, facility managers should acknowledge and recognise LHWs as valuable members of the healthcare team and urge other staff members to do the same. Issues must be resolved equitably and impartially by providing each employee with an equal chance to present their case and refraining from taking sides. It is also important to acknowledge the beneficial experiences that LHWs have gained by working closely with the community while allowing them to suggest ideas for development.

Lay healthcare workers’ voices are reflected below^16,20^:

‘We used to have these meetings, but since 2020, everything changed. Every month we were given a chance to voice our challenges and the clinic staff would do the same. We were not oppressed, like now we are told that we are not tracing properly and the missed appointments are high. They blame us for these high missed appointments whereas they are the ones who do wrong things by not updating files after the clients’ visit to the clinic or obtaining traceable contact details. They point fingers at us, but the other fingers are pointing at them for not updating the files correctly.’ (T2F2D, 28-years-old, female, lay health care worker)^16,20^

‘From my side, I think that if there are suggestions or opinions required, we must be involved and be asked directly. Most of the time they ask the supervisors who do not even see patients. We are the people on the ground working with clients every day and we know the challenges better than the supervisors.’ (T1F1B, 34-years-old, male, lay health care worker)

‘Again, there was someone last time who was informing clients on chronic medication about the survey. I did not know what she was talking about and if I was involved, I could have helped her to better explain to the clients in a way that they would understand. I am just saying that we should be part of anything that patients need to be empowered on from the beginning so that we are well-informed and can help to cascade the information.’ (T1F1F, 59-years-old, female, lay health care worker)^16^

**Step 2:** Identify individual needs of lay healthcare workers: Each LHW’s unique requirements can be determined either verbally by the LHWs or through analysis by the supervisor. To evaluate the supporting behaviours, LHWs and supervisors can collaborate to create support checklists.^16^ Additionally, supervisors and LHWs can collaborate to create personal development plans and track progress. Updated information, refresher training, orientation, supervision, debriefing and improved stipends are a few examples of needs verbalised by LHWs, as discussed under the recipient. By taking this step, the agents can facilitate support that is tailored to the requirements that have been identified, which will boost the LHWs’ sense of assistance and satisfaction. The different needs of LHWs are highlighted by the quotations below^16^:

‘I wouldn’t say I know how to communicate effectively with a client who does not open up, so I just leave him/her.’ (T2F2D, 28-years-old, female, lay health care worker)

‘But going back to the non-disclosure issue, it is a challenge, and it is difficult for me as well. It is not safe for us because sometimes the couple starts to argue. We just don’t know how to deal with non-disclosure among couples.’ (T3F3C, 36-years-old, female, lay health care worker)

‘It is affecting performance negatively. For example, we were told to strengthen viral load education, but since only certain lay counsellors from developmental partners were given demonstration bottles, then it means we must refer all clients to them. How are we going to educate clients if we do not have the resources?.’ (T3F3B, 48-years-old, male, lay health care worker)

‘Also, in most cases when there are workshops, counsellors attend, and I am left behind. I only receive brief feedback, so how am I going to provide information to clients if I am not trained? This is not nice at all. Like index programme, I do not understand it, no one trained me. If I assist with testing and get an HIV positive client, I have to run around to look for a counsellor to assist me because I do not have a clue of what they say or how they offer index testing.’ (T1F1, 59-years-old, female, lay health care worker)

**Step 3:** Provide emotional and instrumental support: Based on the needs that have been identified and the demands of the job, healthcare professionals can continue to provide support. LHWs described emotional support in examples: help with challenging clients, resolving problems, equitable distribution of resources that prevent discrimination, setting up debriefing sessions and meetings to talk about challenges in the field, and appreciation.^16^ In addition, the department of health can enhance employment conditions by providing burial plans, bonuses and increases in stipends. Actual assistance with client care, for example, nurses helping working clients promptly; the provision of refresher courses and updated HIV information, such as new ART, working material like HIV test kits, gloves, and visual educational aids that promote adherence counselling; identification of implementation gaps like the use of HIV guidelines and subsequent mentoring; job-orientation for new LHWs; and supportive supervision are examples of instrumental support cited by LHWs.

The voices of some participants are reflected below^16^:

‘The salary issue is a sad one, but I still treat my patients well. We have been working for a long time but there is nothing to show for it. If I happen to die, my family is the one that will make arrangements for me to get home (mentioned the place ± 100km away). There won’t be any cent from my workplace, my kids will not get anything. Yes, I continue to help patients, but I am sad.’ (T3F3B, 48-years-old, male, lay health care worker)

‘We are not debriefed regularly. The last time I was debriefed was in 2020 after demanding it. In most cases, tracers are not included in debriefing activities, only counsellors are.’ (T4F4A, 30-years-old, male, lay health care worker)

‘And actually, we do not require a big thing, if we can just meet as colleagues, have a professional person and then talk about our work and how to cope, it will be good.’ (T4F4E, 42-years-old, female, lay health care worker)

‘In terms of the other support, my supervisor provides support through provision of working material and stationery.’ (T4F4D, 28-years-old, male, lay health care worker)

‘We need consistent in-service training. Our information becomes outdated, and we also do not practise some skills like adherence counselling, so we forget. So, refresher training is important.’ (T2F2C, 44-years-old, female, lay health care worker)

**Step 4:** Foster mutual dependence: To improve retention in HIV care and other health outcomes, managers need to foster collaboration among all facility workers by outlining roles, sharing responsibilities and elucidating how all operations are interconnected. The LHWs emphasised the value of collaboration and knowing what their co-workers are working on. They emphasised how crucial it is for clinic employees to record and maintain accurate client contact information, which helps with tracing, and to connect clients with CHWs so that they may receive regular visits.^16^ Furthermore, lay counsellors should step up their counselling and education efforts as soon as an HIV diagnosis is made, and they should also stop regarding retention as the exclusive purview of CHWs and tracers. Additional concerns that need to be addressed to reduce frustration and treatment interruption by clients are the negative staff attitudes and the loss of clients’ medical files.

Participants said^16^:

‘We request OPMs (operational managers), counsellors, admin clerks and all other staff members to improve communication. The communication must be on point and there must not be communication breakdown. Counsellors must also help us by explaining to clients that the CHWs assist the elderly client with treatment collection and not everyone. Youth must be aware that the CHWs are not responsible for delivery of medication to everyone. This information must also be spread in the adherence clubs. The files must be checked and updated before the CHWs can be sent for tracing.’ (T2F2F, 22-years-old, female, lay health care worker)

‘I suggested to the facility manager to help tracers and CHWs to meet and also guide us in terms of how we can work harmoniously together. I also work for the developmental partner, and it is difficult for me to give suggestions directly to my colleagues. So, the facility manager drafted a weekly schedule of physical tracing and we have been following it. The only challenge is the CHWs who are working at the vaccination sites, but we are currently working with those available. Now I know that when I have challenges, I report to the facility manager who in turn communicates with the outreach team leader. So far there is good progress.’ (T4F4B, 31-years-old, female, lay health care worker)

‘Teamwork from colleagues. If someone is good or knowledgeable, he/she must assist those that are struggling so that we can improve. We must be aware of each other’s activities as lay health care workers so that we can understand how our work is interrelated and work effectively.’ (T2F2A, 33-years-old, female, lay health care worker)

‘So, we really need to think of a way to encourage clients to provide correct contact details. This is where we really need to synergize our efforts from counsellors, clerks, tracers, CHWs and nurses. We must all be involved.’ (T4F4E, 42-years-old, female, lay health care worker)^16^

**Step 5:** Provide constructive feedback: Two-way client referrals between LHWs and facility personnel, LHWs’ participation in staff meetings, and politely discussing performance gaps and issues without degrading or accusing others are ways to promote constructive feedback.^16^ In addition, individual LHWs can receive feedback on both their good performances and areas for improvement to help them stay motivated and help with future judgements on the necessary support. If a correction is necessary and urgent, supervisors must use caution while handling LHWs and refrain from making corrections in front of clients to avoid embarrassing the LHWs. The following quotes highlight the LHWs’ desire for constructive feedback rather than condemnation^16,20^:

‘Yes, she said it all. There is no time for feedback and this is really affecting our work negatively. We are just working, not knowing if we do the right or wrong things [*others agreed: “yes”*].’ (T2F2F, 22-years-old, female, lay health care worker)

‘Indeed, communication is a problem. Sometimes as a CHW I refer a client to the facility and when the client gets to the facility, the staff informs her/him that they do not know me (Other participants concurred). So how is the community going to trust us, CHWs, if the clinic staff deny knowing us? We tell the community that we work for the Department of Health and indicate the name of the clinic, but the clinic staff say they do not know us. Do you think that when I go back to the same patient, he/she will believe me when I say I work at the particular clinic? We refer clients expecting to get a back-referral (feedback) so that I can get a copy and have evidence that I did my job, but this is not happening.’ (T2F2D, 28-years-old, female, lay health care worker)^20^

‘It angers me because they expect miracles from us. And no matter how hard we work, no one appreciates or commends us for our hard work (Other participants agreed: yes). Instead, they keep on putting pressure on us, they complain and complain. And when they do this, we end up being demotivated and discouraged to work, what is the use of trying to do your best when no one notices or appreciates? A “thank you’ once in a while will do.’ (T2F2A, 33-years-old, female, lay health care worker)^20^

**FIGURE 2:**
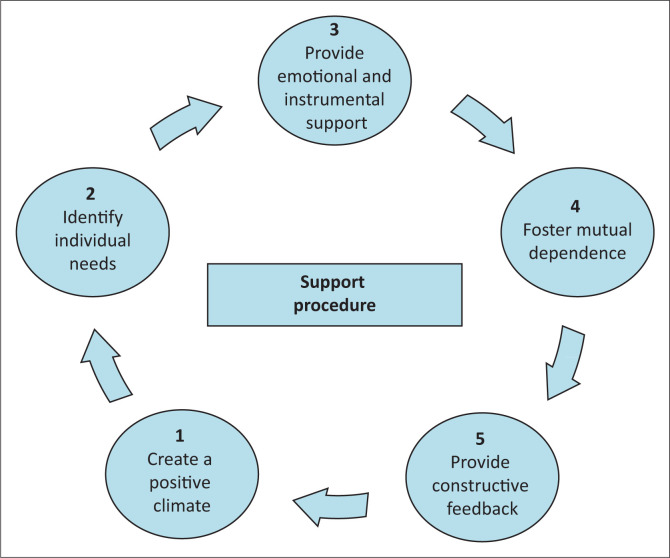
Support procedure.

The ongoing nature of the support procedure is seen in Figure 2.

#### Outcome

**FIGURE 3:**
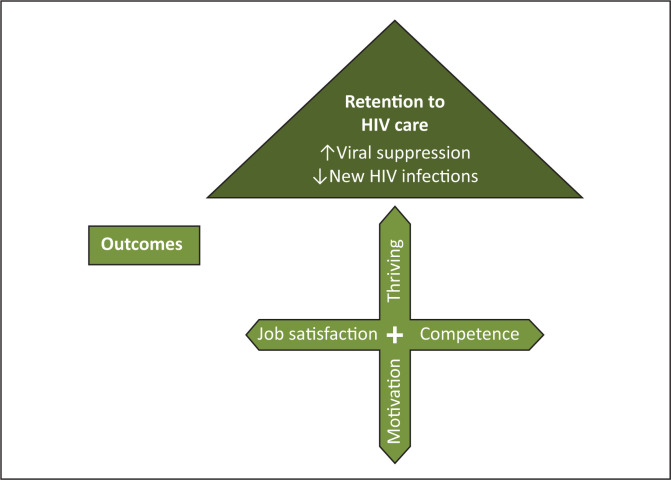
Support outcomes.

The end results of an action are referred to as the outcome. As shown in Figure 3, providing support will potentially improve the motivation, job satisfaction, competence and thriving of LHWs. This could enhance client retention in HIV care and viral suppression, which could lead to a decrease in new infections. The quotations below suggest that the LHWs believe that training, mentoring, feedback, active involvement and better working conditions could improve their motivation levels and skills^16^:

‘It would be better if we had a day, say Tuesday maybe, where we remain in the clinic and not go to the field. We would then reflect, discuss and assist each other and improve our work.’ (T2F2B, 25-years-old, female, lay health care worker)

‘I am not trained to pick up warning signs of mental health issues. And like my colleague said, I never thought it is important. And yes, we see challenging situations at the households, but I normally inform the clinical mentor if I see a client who cannot walk or has social problems. Nothing around mental health issues but I would really love to be trained if there is such a training as this will improve my skills. I am very happy about this interview, sometimes we just wish to have someone who can just listen to us narrating our field work. Otherwise, we just bottle our feelings inside which is not right.’ (T4F4A, 30-years-old, male, lay health care worker)

‘It is the same as index, it is only now that they want us to do it. Sometimes I refuse and let the lay counsellors from the developmental partner continue with it, isn’t it that they started it without involving us? But again, the salary issue is adding to it, I would also like to earn a better salary and be like them, maybe I will be motivated. This issue of the low salary is sad. Sometimes we contribute to the patient’s failing treatment because we help them with heavy hearts, we have anger due to being ill-treatment and we take it out on them, we are just tired. I just wish this salary issue could be resolved.’ (T3F3A, 37-years-old, female, lay health care worker)

‘This is where the problem is. There is no time for meetings in our facility. I believe that we must have monthly meetings with the managers to discuss our challenges, but this is not happening because they are always busy. And I believe that they must make time for us because we have challenges, we are burdened. And how will I do my work effectively when I feel burdened? No, I will not be effective. It is clear that if I am not happy, there will not be production.’ (T2F2C, 44-years-old, female, lay health care worker)^16^

### Relationship statements

The following statements explain how the relationships between the concepts were created^16^:

- Support is an interdependent activity that calls for emotional stability, good relationships, willingness and communication between the recipient and agent, or the LHWs and their supervisors, healthcare professionals and the Department of Health.
- The Department of Health, other organisations and supervisors need to create a safe environment for the LHWs to verbalise their needs for support and perform their work freely without fear of being judged.
- The exchange of support between the LHWs and supervisors may occur in varied settings, such as primary healthcare facilities, mobile clinics, hospitals, the community and non-governmental organisations.
- To enable the support procedures, the dynamics of willingness, communication, emotional stability and positive connections are required.
- Enhanced motivation and competence among LHWs, enhanced retention, viral suppression and decreased rates of new HIV infections are outcomes that will be attained as a result of the support.
- Despite the possibility of positive results, the dynamics and support procedures could be jeopardised by factors that negatively impact LHWs’ attempts to keep clients in HIV care. These factors include lost medical records, unfavourable staff attitudes towards clients, a lack of cooperation, gratitude, and recognition for LHWs, an increase in workload, and unfavourable work contracts.^16^

### Description of the support model

Chinn et al. advocated that the purpose, structure and assumptions of the model can be used to characterise it.^28^

#### Purpose

This model is intended to help healthcare organisations, professionals and supervisors support LHWs in enhancing patient retention in HIV care.

#### Structure

**FIGURE 4:**
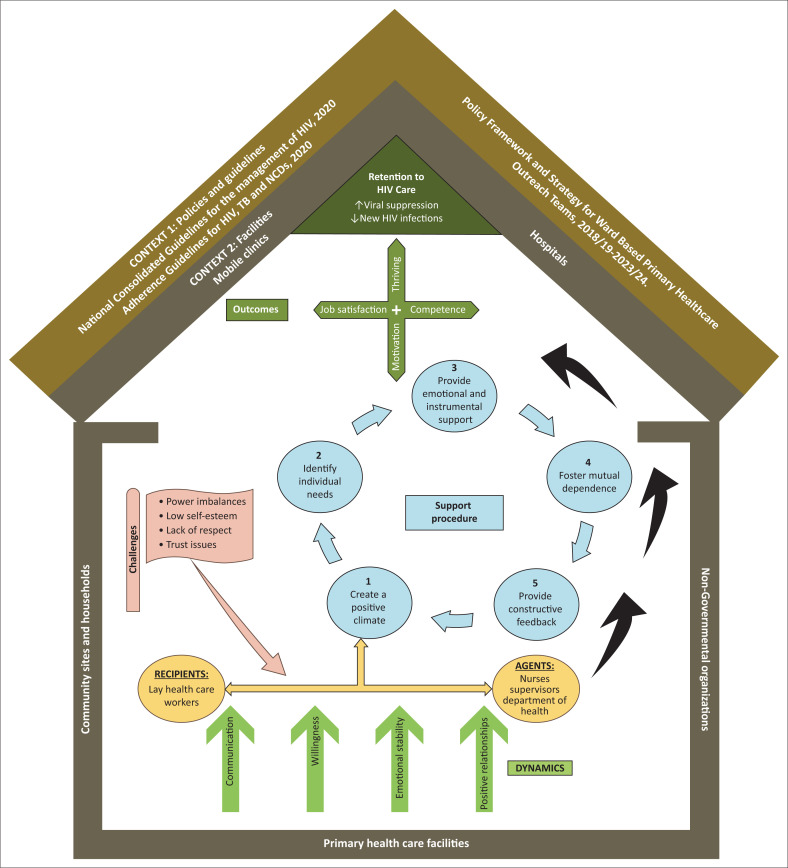
An integrated support model for lay healthcare workers to improve retention in HIV care.

The model is represented in Figure 4 and has six components: context, agent, recipient, dynamics, procedures and outcome. The symbolic significance of each element is:^16^

- The basic design is similar to that of a house, with brown outer walls representing the context or structure. The house serves as a refuge, showing that both a sense of safety and belonging and a favourable environment are necessary for effective interactions.
- Green is considered the colour of hope; hence, the dynamics, which are represented by upward light green arrows, are at the base to demonstrate that they form a basis for helpful interactions.
- Agents and recipients are represented by light orange oval shapes and joined by a two-way arrow to show the interactive nature of support.
- Obstacles are depicted as a pink flag and arrow on the left side to warn organisations that if not addressed, support will be ineffective.
- The support procedures are depicted as a light blue cyclic shape, illustrating how the support actions that aid in retention are ongoing and connected. Retention in HIV care efforts would be hampered if any one or more of these processes were not carried out. It does not, however, imply that every step must happen in the order listed; some, like providing assistance and encouraging mutual dependency, might happen simultaneously or before others.
- The outcomes are displayed at the top in a dark green colour to indicate fulfilment for both the LHWs and the healthcare system. The retention outcome is at the apex of the triangle, and the motivation, work satisfaction, competence and thriving outcomes of the LHWs form a cross directly below the retention outcome.
- Lastly, the black arrows pointing upward on the right side indicate the intended direction of the flow, which begins with the dynamics and continues through to the results.^16^

#### Assumptions

The acknowledged facts that form the foundation of the model are known as assumptions and are connected to the relationship statements.^21^ The following assumptions are derived from Lakey and Cohen’s descriptions of stress and coping, social constructivist and relational viewpoints^29^:

- According to the stress and coping perspective, providing support can help individuals manage their stress levels, and the sense of accessible support can lead to a less negative interpretation of frightening situations.
- The perspective of social constructivism postulates that individuals form conceptions of the world that mirror their surroundings and that diverse individuals or groups may not reach a unanimous agreement on what support entails. Moreover, the social environment supports individuals’ well-being by guiding them to self-regulate and retain their identity and self-esteem based on group standards.
- The relationship viewpoint makes the assumption that high social support is correlated with the quality of interpersonal interactions. In contrast to negative connections, which are marked by criticism, disagreements, battling over scarce resources and violating promises, positive relationships are characterised by sharing, low conflict and social skills.^29,16^

Extracts from the LHWs’ quotations below affirm the last two assumptions discussed above^16^:

‘Yes, communication is really important. Without communication, nothing will improve.’ (T2F2A, 33-years-old, female, lay health care worker)

‘This [*meetings and feedback*] can work for us because our activities are interrelated, and we depend on each other to be successful in improving retention.’ (T1F1F, 59-years-old, female, lay health care worker)

## Critical reflection of the support model

To determine the model’s relevance, clarity, generality, simplicity and accessibility, a critical evaluation is required.^28^ While there was no independent expert evaluation, these criteria are covered in the section that follows.^16^ Three examiners and the researcher’s supervisor also assessed the model and made recommendations based on their evaluations.

### Clarity

The model’s comprehension was enhanced by adhering to the same recognised notions rather than introducing new ones and categorising these concepts using the explicit Dickoff et al. processes, as reported by Dube et al.^21^ and the Chinn et al. model development procedures.^17^

### Simplicity

The model was created with the intention of simply reflecting all of its constituent parts, their interrelationships, motivating factors, difficulties and results.

### Generality

This model can be used to help LHWs’ HIV retention efforts in other provinces of South Africa and other nations with comparable contextual characteristics, even though it was created in the setting of a primary healthcare facility, one of the biggest in the province of North-West. It can also make it easier to sponsor healthcare programmes that LHWs participate in, outside of HIV.

### Accessibility

The model’s accessibility will be enhanced through the writing of a thesis, its uploading to the university’s repository, the submission of papers, presentations at conferences or summits focused on quality improvement, and the submission of a copy of the study results to the Department of Health.

### Importance

The model holds great significance as it possesses the ability to tackle the recognised issue of insufficient assistance for LHWs. This, in turn, can enhance their abilities and motivation, ultimately leading to better outcomes such as enhanced retention in HIV care, suppression of viral load, and a decrease in new HIV infections.

## Discussion of findings

Six key elements were identified in the support model: context, agent, recipient, dynamics, procedure and outcomes. The setting in which the LHWs operate and the policies that direct their work are both included in the context. Although the study included LHWs linked to a government primary healthcare facility to provide on-site and community outreach services, LHWs work in various environments such as government-owned and privately-owned clinics, health centres and hospitals, as well as non-governmental organisations.

The entry point for clients with chronic illnesses and tuberculosis is the LHWs, such as lay counsellors and CHWs, who offer HIV testing, health education and adherence counselling.^30,31^ In addition, the reorganisation of primary healthcare resulted in the extension of services to non-profit and community-based organisations that use LHWs to deliver healthcare. Despite the LHWs not having a formal and regulated career path in South Africa, the national policy and guidelines on HIV,^22,23^ adherence for HIV, TB, and non-communicable diseases,^24^ and policy framework and strategy for ward-based primary healthcare outreach teams^25^ guide HIV care, retention and CHWs’ outreach services. These guidelines are appropriate in both the public and private sectors and are periodically updated in light of new research findings.

Moreover, certain dynamics, namely, communication, willingness, emotional stability and positive relations, serve as a good foundation for supportive actions between the agents (healthcare professionals) and recipients (LHWs).^16^ However, they are hindered by power imbalances and lack of respect and trust, which may result in LHWs’ low self-esteem. Costa et al. affirmed that in the absence of trust, teamwork is hindered as workers withhold their important contributions.^32^ Again, Ludwick et al. stated that managers and healthcare workers must develop strategies to foster positive interactions and connections, such as referral mechanisms and respect, to contribute to enhanced CHW programme outcomes.^33^ Also, Kok et al. suggested clear operating procedures and rules, as well as clearly defined duties and associations with other healthcare professionals, in order to improve the efficacy of CHWs,^34^ while Scott et al. hinted that integration that fostered civil cooperation and communication among healthcare workers inspired and stimulated the CHWs to impart their special, applied knowledge, thereby enhancing client care.^35^

Establishing the aforementioned dynamics and being cautious of challenges that hinder them, paves a way for five cyclic steps of the support procedure, which may result in improved LHWs’ motivation, competence, job satisfaction, retention in HIV care and programme outcomes.^16^ These steps are as follows: creating a good environment, identifying individual LHWs’ needs, offering both instrumental and emotional support, encouraging mutual dependency and providing feedback. However, these steps may not always be completed in the given order, and some might also be carried out simultaneously. A good environment for LHWs is supported by Busza et al., who revealed that the CHW programmes are useful in enhancing HIV services and adherence to treatment, but their impact depends on establishing supportive work settings for CHWs.^36^

The importance of assessing the individual LHWs’ needs was supported by Caesens et al., who mentioned that as intensive support may be interpreted as incompetence or may lower self-esteem in persons with low socio-economic requirements, customised supportive interventions are key.^37^ Similarly, Hodgins et al. advocated for LHWs to receive individualised support to effectively implement health programmes.^15^ In terms of the actual support, LHWs identified instrumental support such as information, assistance with clients and provision of working resources, as well as emotional support, for example, respect, appreciation, debriefing, better salary and work contracts. According to Mathieu et al., instrumental support consists of task assistance, information and other concrete elements, which are consistent with the examples given by LHWs. Emotional support, on the other hand, involves listening to people and demonstrating regard, encouragement, compassion and sympathy.^38^ Moreover, even if the healthcare system is still having challenges with controlling and incorporating LHWs, interventions such as follow-up, training, monitoring and debriefing can make them feel valued as members of the team, which will improve their morale and the calibre of counselling they provide.^39^

The LHWs also emphasised the need to improve staff attitudes, record and update client contact information and clinic visits, and improve the filing system to reduce the loss of medical records, which can cause patients to become unsatisfied and interrupt treatment. Maughan-Brown et al. concurred that low client retention in care was partly caused by administrative errors, such as misplacing medical data.^40^ Furthermore, Scott et al. found that polite cooperation and communication between CHWs and other healthcare workers encouraged the CHWs to impart their special, useful expertise, improving patient care.^35^

Benefits of debriefing as an emotional support method include reduced errors, increased job satisfaction and improved transparency in communication.^41^ Again, according to Jacobs et al., lay counsellors were content with debriefing-on-the-go, in which they would get together once a week to discuss challenging situations, exchange experiences, role-play, and devise better methods to approach issues.^42^ In terms of instrumental support, Letsoalo et al. discovered that while some lay counsellors began their jobs without formal training, those who did still needed refresher courses to remind them of some aspects of their work that they might have forgotten.^11^ Likewise, Bemelmans et al. discovered that on-the-job coaching and training in adapted and disclosure counselling are necessary to enhance lay counsellors’ capacity to handle new responsibilities.^43^

In addition, Mundeva et al. advised that to prevent CHWs from burning out, they should receive continual training and supervision while working in HIV care and other programmes.^8^ Assegaai et al. further emphasised that CHWs’ performance can be enhanced by providing them with basic training and on-the-spot assistance.^44^ Once more, the lay counsellors believed that using counsellor-client role plays and group monitoring enhanced their abilities and self-assurance.^42^ Similarly, Schmitz et al. discovered that supportive supervision, integration of LHWs’ activities into public health systems and appropriate compensation can inspire LHWs, which in turn can result in high-quality, effective interventions.^45^

Another study that evaluated the psychosocial difficulties faced by HIV counsellors identified four areas of support: supervision to identify and address service delivery gaps; management support, where counsellors voice concerns and offer suggestions for enhancements; emotional support through debriefing or the identification of healthy coping mechanisms and ongoing in-service training with updated HIV information.^46^ Kok et al. further established that the context, the health system and intervention hardware, and the health system and intervention software are the other three kinds of elements that affect the CHWs’ functioning.^10^ In addition, software elements include ideas, interests, relationships, power and norms of the health system factors that influence CHWs’ feelings of self-fulfilment, familiarity and connectedness, as well as perceptions of support, respect, competence, honesty, fairness and recognition – all of which would improve CHWs’ performance if strengthened. Hardware elements include supervision systems, training, accountability, communication structures, incentives, supplies and logistics.^10^

### Strengths and limitations

The study created a novel paradigm that emphasises the importance of support and how various lay healthcare workers’ activities in HIV care and retention are inherently interconnected and vital. Though the methods and context were provided to aid replication, the study was carried out in a community health centre in the Bojanala area and may not be generalised to all districts, provinces or nations. In addition, the results were contrasted with those from other nations and provinces. The researcher also notes that not all of the literature on retention and support was reviewed; still, pertinent publications were chosen, and data saturation – the point at which fresh or novel data stopped emerging – was taken into account.

## Recommendations

Based on the findings, we recommend the following for future research:

- Studies on patient retention or disengagement from care patterns have been conducted; however, because of significant client mobility and duplication at several healthcare facilities, the results have occasionally presented a skewed picture. As there is currently no centralised HIV care database, future research may concentrate on methods that enable clients to minimise both false and true loss to follow-up.
- Also, there is a need to look into the expenses associated with setting up electronic medical records, particularly for institutions that handle a lot of patients.^16^

Our recommendations for practice, include:

- Equal training on adherence counselling is necessary for all LHWs because they will need to employ these abilities at some stage. Again, lay counsellors should not be the only LHWs to receive debriefing; creative and affordable solutions can be considered to debrief all categories of LHWs.
- Provide information and instructional resources to all lay counsellors without bias, to support high-quality patient care and project longevity. This can be done by unifying the supervisory activities of supervisors from the department of health and the development partner.^16^
- Respect, cooperation and feedback are aspects that managers and supervisors should promote because retention activities are connected.
- Managers should monitor compliance with the national filing requirements to minimise the loss of clients’ files.
- Supervisors need to monitor the application of HIV and adherence standards and work on any gaps. In addition, they must evaluate each LHW’s sense of assistance by utilising the existing checklists or customising them, and then respond to the requirements identified.^16^

We recommend for policy and governance the following:

- Given their critical role in HIV management, the Department of Health should investigate the possibility of officially acknowledging LHWs as members of the health workforce. This might involve updating their pay and benefits and creating a formal training programme for them.^16^
- It is necessary to reassess the HIV testing targets for lay counsellors so that they can devote more time to adherence activities. The adherence standards specify the steps to be taken in the event that a client interrupts his or her treatment; nonetheless, tracing tools or checklists that instruct tracers on how to effectively communicate with clients who have interrupted treatment are still needed.
- It is imperative to enhance mental health services by implementing screening checklists and providing LHWs with suitable training to enable them to recognise warning signals and make necessary referrals, as mental health concerns are adversely affecting retention.
- Moreover, given that the facility has over 7 000 clients on ART, it is necessary to look at file loss at the facility and research workable solutions.^16^

## Conclusion

In keeping with the goal, an integrated support model was created for lay healthcare workers to help them better retain clients in HIV care. In order to support LHWs’ knowledge, abilities, motivation and job satisfaction, support is a resource that must be given consistently. Collaboration and mutual reliance between lay and professional healthcare workers are necessary for HIV care retention. The created model has the potential to enhance the assistance given to LHWs in other programmes as well as HIV care.
